# Supplementary material for: WordCommentsAnalyzer: A windows software tool for qualitative research
Source: F1000Res. 2018 Sep 4;7:536. Originally published 2018 May 3. [Version 2] doi: 10.12688/f1000research.14819.2 (PMC6137411; doi:10.12688/f1000research.14819.2)
Supplement: Supplementary file 3 [file f1000research-7-17614-s0002.tgz › b2a79496-4ada-47c4-9682-a99ce867836d.zip!/Eight Iranian Health Journals/Archives of Rehabilitation_32.docx]

© Archives of Rehabilitation. This work is licensed under CC BY-NC 4.0 (https://creativecommons.org/licenses/by-nc/4.0/).

Volume & Issue: دوره 12 - شماره 3

Title: مقایسه نتایج یک آزمون عملکرد-محور با دو پرسشنامه خود-اظهاری در تعیین میزان ناتوانی بیماران مبتلا به کمردرد مزمن

http://rehabilitationj.uswr.ac.ir/article-1-643-fa.html

مقدمه: پرسشنامه‌های خود-اظهاری و آزمون‌های عملکرد-محور دو شیوه ارزیابی میزان ناتوانی در انجام عملکردهای روزمره بیماران مبتلا به کمردرد مزمن هستند. هدف این مطالعه بررسی نحوه اثرگذاری درد بر میزان ناتوانی این بیماران از طریق مقایسه یافته‌های حاصل از پرسشنامه‌های خود-اظهاری رولندموریس و اسوستری با آزمون عملکرد محوری بنام «مقیاس عملکردی کمر» بوده است. روش بررسی: پژوهش حاضر یک مطالعه مقطعی و جامعه آماری بیماران مبتلا به کمردرد مزمن بدون علت خاص می‌باشند.75 بیمار 60-18 ساله به روش نمونه‌گیری آسان وارد مطالعه شدند. مقیاس عملکردی کمر و پرسشنامه‌های رولندموریس و اسوستری برای ارزیابی ناتوانی و شدت درد با VAS اندازه‌گیری شد. ارتباط بین دو متغیر شدت درد و ناتوانی به کمک ضریب همبستگی اسپیرمن و ارتباط بین شیوه‌های مختلف ارزیابی ناتوانی با محاسبه ضرایب همبستگی پیرسون تعیین گردید. تفاوت میزان ناتوانی در دو گروه دارای درد خفیف تا متوسط و گروه درد شدید با آزمون تی مستقل مقایسه شد. یافته‌ها: ضرایب همبستگی بین VAS و مقیاس عملکردی کمر، پرسشنامه‌های رولندموریس و اسوستری به ترتیب 0.37، 0.55 و 0.54 (0/01>P) و ضرایب همبستگی بین مقیاس عملکردی کمر و پرسشنامه‌های رولندموریس و اسوستری به ترتیب 0.41 و 0.52 (0/001>P) به دست آمد. مقیاس عملکردی کمر تفاوت معنی داری را بین میزان ناتوانی در دو گروه درد نشان نداد (0/139=P) اما این تفاوت در نتایج پرسشنامه‌ها معنی دار شد (0/01>P). نتیجه‌گیری: عدم تشابه بین نتایج پرسشنامه‌های خود-اظهاری با مقیاس عملکردی کمر و همبستگی متوسط بین آنها نشان می‌دهد که دو شیوه ارزیابی می‌توانند جنبه‌های مختلفی را در فرآیند ناتوانی نمایان سازند؛ لذا برای ترسیم نمایی جامع از وضعیت عملکردی بیماران استفاده توأم از هر دو شیوه ارزیابی توصیه می‌گردد.

Title: بررسی تکرارپذیری اندازه‌گیری دامنه حرکتی ناحیه کمر با استفاده از تست دو بار اصلاح‌شده شوبر در افراد سالم

http://rehabilitationj.uswr.ac.ir/article-1-278-fa.html

هدف: اندازه‌گیری درست دامنه حرکتی ناحیه کمر هم در ارزیابی بیماران و هم در سنجش اثر درمان‌های مختلف توانبخشی از ارزش بالایی برخوردار است. هدف مطالعه فوق بررسی تکرارپذیری تست دو بار اصلاح‌شده شوبر (Modified-Modified Schober's Technique) در اندازه‌گیری دامنه حرکتی ناحیه کمر توسط آزمون‌گر واحد می‌باشد. روش بررسی: در یک مطالعه متدولوژیک، دامنه حرکتی ناحیه کمر (خم‌شدن به جلو و عقب) ۳۸ فرد سالم (۱۲ مرد و ۲۶ زن) بدون سابقه کمردرد، توسط تست دو بار اصلاح‌شده شوبر در سه نوبت اندازه‌گیری شد. دو اندازه‌گیری در یک روز به فاصله یک ساعت برای بررسی تکرارپذیری درون-روز (Within-Day) و نوبت آخر با فاصله یک هفته برای ارزیابی تکرارپذیری بین-روزها (Between-Days) صورت گرفت. یافته‌ها: اندازه‌گیری حرکت خم‌شدن به جلو و عقب درون روز تکرارپذیری بالایی را نشان می‌دهد (۰/۸۵=ICC برای اندازه‌گیری خم‌شدن به جلو و ۰/۷۳=ICC برای اندازه‌گیری خم‌شدن به عقب). نتایج همچنین ضریب همبستگی قابل قبولی را برای تکرارپذیری اندازه‌گیری حرکت خم‌شدن به جلو و عقب بین روزها را نشان داده است (۰/۸۳=ICC برای اندازه‌گیری خم‌شدن به جلو و ۰/۶۸=ICC برای اندازه‌گیری خم‌شدن به عقب). نتیجه‌گیری: نتایج مطالعه حاضر نشان می‌دهد که تست دو بار اصلاح‌شده شوبر از تکرارپذیری بالایی در اندازه‌گیری دامنه حرکتی ناحیه کمر برخوردار بوده و می‌تواند به عنوان یک ابزار قابل اعتماد در ارزیابی بیماران و همچنین در بررسی اثر مداخلات مختلف درمانی به‌کار گرفته شود.

Title: مقایسه تأثیر دو برنامه فعالیت ورزشی هوازی تداومی و تناوبی منتخب بر ظرفیت عملکردی بیماران پس از جراحی پیوند عروق اکلیلی

http://rehabilitationj.uswr.ac.ir/article-1-454-fa.html

هدف: تمرین ورزشی انواع مرگ‌ومیرها و همچنین مرگ‌ومیر ناشی از بیماری‌های قلبی‌عروقی را با بهبود ظرفیت عملکرد قلب بیماران کاهش می‌دهد. هدف از انجام این پژوهش مقایسه تأثیرات تمرین‌های ورزشی تداومی و تناوبی هوازی پرشدت بر ظرفیت عملکردی بیماران قلبی متعاقب جراحی پیوند عروق اکلیلی است. روش بررسی: در این مطالعه تجربی و مداخله‌ای، 33 بیمار جراحی پیوند عروق اکلیلی (30 مرد و 3 زن) مطابق معیارهای لازم و به صورت در دسترس انتخاب و در سه گروه برنامه تمرینی تداومی هوازی (۱۲ نفر) شامل 30 تا 60 دقیقه فعالیت تداومی با شدت 70 تا 85 درصد حداکثر ضربان قلب، برنامه تمرینی تناوبی هوازی (۱۲ نفر) شامل 28 تا 44 دقیقه فعالیت تناوبی با شدت 75 تا 90 درصد ضربان قلب -در قالب 3 دوره به همراه 5 تا 10 دقیقه استراحت بین هر دوره، حداکثر به مدت 8 هفته و هر هفته 3 مرتبه- و گروه کنترل (۹ نفر) قرار گرفتند. ارزیابی‌های لازم قبل و بعد از برنامه ورزشی از طریق اندازه‌گیری ظرفیت عملکردی با استفاده از تست ورزشی (پروتکل بروس اصلاح شده) انجام شد. آزمون‌های آماری t زوجی و تحلیل واریانس یک‌سویه به همراه آزمون تعقیبی دانت برای تجزیه تحلیل یافته‌ها استفاده شد.  یافته‌ها: بین ظرفیت عملکردی دو گروه تمرین تداومی و تناوبی بعد از انجام برنامه، تفاوت معناداری وجود نداشت. مقایسه ظرفیت عملکردی بعد از برنامه بین گروه‌های تمرینی (تمرین تداومی و تناوبی) و گروه کنترل، تفاوت معناداری را نشان داد (۰/۰۱۷= P). مقایسه دقیق‌تر نشان‌دهنده وجود تفاوت معنادار ظرفیت عملکردی بین گروه تداومی و کنترل (۰/۰۰۹= P) و عدم وجود تفاوت معنادار بین گروه تناوبی و کنترل بود. نتیجه ‌گیری: یافته‌های این پژوهش لزوم طراحی هر دو برنامه تمرینی تداومی و تناوبی پرشدت هوازی برای بیماران مبتلا به گرفتگی عروق کرونر و به خصوص بیماران قرار گرفته تحت جراحی پیوند عروق اکلیلی و همچنین بهبود سلامتی و بهداشت بیماران قلبی در آینده را نشان می‌دهد.

Title: ارزیابی عملکرد حلزون شنوایی در شیرخواران و کودکان مبتلا به بیماری مننژیت بر اساس نتایج آزمون گسیل‌های صوتی برانگیخته

http://rehabilitationj.uswr.ac.ir/article-1-453-fa.html

هدف: ارزیابی عملکرد حلزون شنوایی در شیرخواران و کودکان مبتلا به بیماری مننژیت بر اساس نتایج آزمون گسیل‌های صوتی برانگیخته گوشی هدف این مطالعه بوده است. روش بررسی: در این پژوهش تحلیلی–مقطعی، 40 شیرخوار و کودک مبتلا به مننژیت در محدوده سنی بدو تولد تا 12 سال، از بین مراجعین بیمارستان مفید و با انتخاب تمام‌شمار، توسط آزمون گسیل‌های صوتی برانگیخته شامل گسیل‌های گذرا و گسیل‌های محصول اعوجاج جهت ارزیابی عملکرد حلزون شنوایی 7 تا 14 روز پس از بهبودی و ترخیص از بیمارستان– مورد ارزیابی قرار گرفتند. آزمون‏ تی مستقل و آزمون دقیق فیشر جهت تحلیل داده‌ها استفاده شد. یافته‌ها: در 35 بیمار عملکرد حلزون شنوایی کاملاً هنجار و در ۵ بیمار نتایج آزمون گسیل‌های صوتی برانگیخته مؤید آسیب جدی به حلزون شنوایی به صورت آسیب شنوایی حسی–عصبی ( ۴ بیمار در دو گوش و یک بیمار در یک گوش) بود. ارتباط بین وضعیت شنوایی و سن (0/002=P)، اختلاف میانگین سطح قند و پروتئین مایع مغزی–نخاعی بین دو گروه با عملکرد هنجار و ناهنجار حلزون شنوایی (0/02=P) و ارتباط بین جنسیت و وقوع عملکرد ناهنجار حلزون شنوایی و SNHL در نمونه‌های مورد مطالعه (0/03= P ) معنادار بود. نتیجه‌گیری: آسیب به اندام کورتی و ساختارهای حلزون شنوایی، به عنوان یکی از پی‌آمدهای بیماری مننژیت، به کمک نتایج آزمون گسیل‌های صوتی برانگیخته قابل ردیابی است.

Title: مقایسه اثر دو روش جدید و رایج «آموزش در خانواده برنامه توانبخشی مبتنی بر جامعه» بر آگاهی، مهارت و نگرش کارکنان محلی توانبخشی

http://rehabilitationj.uswr.ac.ir/article-1-962-fa.html

هدف: پژوهش حاضر با هدف مقایسه اثر روش جدید «آموزش برنامه توانبخشی مبتنی بر جامعه در خانواده معلولان جسمی-حرکتی» بر آگاهی، مهارت و نگرش کارکنان محلی توانبخشی (توان‌یاران) در مناطق روستایی با برنامه آموزشی رایج انجام شد. روش بررسی: در این کارآزمایی میدانی 34 توان‌یار محلی تازه‌وارد در استان لرستان بر اساس روستای محل اشتغال، به دو گروه 18 و 16 نفره تقسیم شده، سپس هر یک از دو گروه به یکی از دو شیوه دریافت آموزش جدید «تکنیک‌های بارش افکار و ایفای نقش» و رایج «سخنرانی و روخوانی متن کتابچه‌های آموزشی» تخصیص یافتند. در شیوه جدید محتوای کتاب‌های خانواده ساده‌نویسی شده، کتابچه راهنمای آموزشی توان‌یار اضافه شد. آگاهی و نگرش دو گروه قبل و بلافاصله پس از دوره آموزشی با استفاده از پرسش‌نامه‌ای مستخرج از محتوای کتابچه‌های آموزش در خانواده و مهارت ایشان از طریق امتحان عملی مورد سنجش قرار گرفت. برای تجزیه و تحلیل داده‌ها از آزمون‌های آماری تی‌مستقل، تی‌زوجی و من‌ویتنی استفاده شد. یافته‌ها: میانگین نمرات آگاهی و مهارت توان‌یاران آموزش‌دیده به شیوه جدید نسبت به گروه دیگر افزایش بیشتری داشت (P<0/001) و توان‌یاران آموزش‌دیده به شیوه جدید به نسبت بیشتری به مراقبان تحت پوشش خود علاوه بر ارائه آموزش شفاهی آموزش عملی ارائه کرده بودند (P=0/028).اما نگرش دو گروه در مورد اثر بخشی برنامه تفاوت معناداری نداشت. نتیجه‌گیری: از آنجا که آموزش به شیوه جدید در ارتقاء سطح آگاهی و مهارت توان‌یاران محلی تأثیر بیشتری داشته، اما منابع تخصیص یافته تفاوت چندانی نداشتند، برنامه‌ریزی برای جایگزینی شیوه جدید شایسته به نظر می‌رسد.

Title: مقایسه ویژگی‌های شناختی، روانی و اجتماعی والدین کودکان بیش از یک فرزند استثنایی با والدین کودکان عادی

http://rehabilitationj.uswr.ac.ir/article-1-904-fa.html

هدف: پژوهش حاضر به منظور دست یابی به علل و عوامل شناختی، روانی و اجتماعی والدین که احتمالاً در تولد کودکان استثنایی در خانواده‌هایی با بیش از یک فرزند استثنایی مؤثر است، انجام شد. روش بررسی: پژوهش از نوع مقایسه‌ای پس رویدادی است و نمونه‌ای شامل ۸۰ زوج از والدین با بیش از یک فرزند استثنایی و ۵۳ زوج از والدین با فرزند عادی از جامعه آماری شامل کلیه والدین با بیش از یک فرزند استثنایی و والدین با فرزند عادی در شهرستان ارومیه به صورت در دسترس گرفته شد. در پژوهش حاضر از ابزار پرسشنامه ویژگی‌های روانی اجتماعی والدین با بیش از یک فرزند استثنایی و پرسشنامه رضایت زناشویی (Enrich) استفاده گردید. برای تحلیل داده‌ها از آزمون‌های مجذور خی توافقی، یو من ویتنی و آزمون تی استفاده شد. یافته‌ها: در میان متغیرهای شناختی، روانی و اجتماعی، بین متغیرهای شغل (پدر و مادر) (۰/۰۰۱>P)؛ تحصیلات (پدر و مادر) (۰/۰۰۱>P)؛ زمان آگاهی از معلولیت (۰/۰۰۱>P)؛ میزان تمایل به ازدواج والدین (۰/۰۰۱>P)؛ نوع رابطه والدین قبل از تولد (۰٫۰۰۱>P)؛ نوع رابطه والدین هنگام بارداری (۰/۰۰۱>P)؛ سلامت روانی مادر (هر چهار عامل جسمانی (۰٫۰۳۷=P، اضطراب ۰٫۰۴۱=P، عامل اجتماعی ۰/۰۴=P و افسردگی ۰/۰۰۱=P) و رضایت زناشویی (۰/۰۳۰=P) تفاوت معنادار مشاهده گردید. در مورد سایر متغیرها تفاوت معناداری مشاهده نشد. نتیجه‌گیری: پیشگیری و برنامه‌ریزی برای کنترل متغیرهای مورد بحث در تحقیق حاضر شامل زمان آگاهی از معلولیت، میزان تمایل به ازدواج والدین، نوع رابطه والدین قبل از تولد و حین بارداری و نیز سلامت روانی مادر و رضایت زناشویی می‌تواند علاوه بر بهبود وضعیت حاضر این خانواده‌ها، به کاهش بروز چنین خانواده‌هایی کمک نماید.

Title: غربالگری تکاملی کودکان ۶۰-۴ ماهه شهر تهران با استفاده از آزمون‌های غربالگری تکاملی دنور۲ و پرسشنامه سنین و مراحل

http://rehabilitationj.uswr.ac.ir/article-1-682-fa.html

هدف: این مطالعه به منظور بررسی وضعیت تکاملی کودکان ۴-۶۰ ماهه شهر تهران با استفاده از آزمون‌های غربالگری تکاملی دنور۲ (DDST-II) و پرسشنامه سنین و مراحل (ASQ)، مقایسه نتایج غربالگری و تعیین ضریب توافق دو آزمون صورت گرفت. روش بررسی: در این مطالعه مقطعی، ۱۹۷ کودک (۱۰۴ پسر و ۹۳ دختر) ۶۰-۴ ماهه (میانگین ۲۳/۵) به‌وسیله DDST-II و ASQ در چهار مرکز بهداشتی درمانی شهر تهران با استفاده از روش نمونه‌گیری در دسترس، مورد غربالگری تکاملی قرار گرفتند؛ که در ۱۵٪ کودکان، ارزیابی به‌وسیله متخصصین کودکان آشنا به حیطه تکامل – که از نتیجه غربالگری اطلاعی نداشتند – تکرار شد. یافته‌ها: نتایج DDST-II و ASQ به ترتیب ٪۳۷ و ٪۱۸ از کودکان را مبتلا به تأخیر تکاملی نشان داد؛ که با در نظر گرفتن مواردی که علیرغم طبیعی بودن، از نظر والدین مشکوک بودند، این میزان به‌ترتیب به ٪۳۸ و ٪۴۳ می‌رسید. ضریب توافق کاپا بین دو آزمون ضعیف بود (۰/۲۱) (۰/۰۰۲=P) ( حیطه حرکات درشت ۰/۲۴، گفتار و زبان ۰/۱۸، فردی- اجتماعی ۰/۰۶ و حرکات ظریف ۰/۰۵). ضریب توافق بین معاینه پزشک با DDST-II و ASQ به ترتیب ۰٫۱۷ (۰٫۰۹۹=P) و ۰٫۴۷ (۰٫۰۰۳=P) بود. نتیجه‌گیری: این مطالعه مشخص کرد که ضریب توافق بین نتایج آزمون‌های غربالگری DDST-II و ASQ ضعیف است، بنابراین برای انتخاب یک ابزار غربالگری مناسب، باید نتایج حاصل از هر دو آزمون با یک آزمون استاندارد طلایی تشخیصی مقایسه شوند.

Title: بررسی انواع فلج مغزی و اختلالات همراه در مناطق شمال و شرق تهران

http://rehabilitationj.uswr.ac.ir/article-1-683-fa.html

اهداف: فلج مغزی، اختلال و ناهنجاری غیر پیش‌رونده در مغز در حال رشد است و یکی از علت‌های اصلی و رایج اختلالات تکاملی دوران کودکی است. این مطالعه به منظور شناسایی انواع فلج مغزی و اختلالات همراه با آن صورت گرفت. روش بررسی: در این مطالعه کودکان ۱ تا ۶ ساله مناطق شمال و شرق تهران با تشخیص فلج مغزی بررسی شده و انواع آن بر اساس طبقه‌بندی اروپایی فلج مغزی (SCPE) طبقه‌بندی گردید. شرکت‌کنندگان توسط تیم پزشکی و توانبخشی مورد ارزیابی قرار گرفته و اختلالات همراه بر حسب وجود علائم صرع، اختلالات گفتار و زبان، مشکلات بینایی، اختلالات شنوایی، و تأخیر در رشد شناختی دسته بندی گردید یافته‌ها: در طول دوره مطالعه ۱۱۲ کودک با فلج مغزی شرکت داشتند. میانگین سنی در پسران برابر با ۳۳/۳ و برای دختران ۲۴/۸ ماه بود. شیوع فلج مغزی در این کودکان در نوع اسپاستیک یک طرفه ٪۳۶/۶، اسپاستیک دو طرفه ٪۴۳/۷ (دایپلژی ٪۳۲/۲ و کوادری پلژی ٪۱۲/۵)، آتاکسیک ٪۱۳/۴ و دیس کینتیک ٪۴/۵ موارد گزارش گردید. اختلالات گفتار و زبان ٪۳۳، صرع ٪۳۰/۴، استرابیسم ٪۱۶/۱، اختلال شنوایی ٪۸ و کم توانی ذهنی ٪۷/۱ مشاهده گردید. نتیجه‌گیری: در این مطالعه فلج مغزی اسپاستیک یک طرفه شایع‌ترین نوع فلج مغزی مشاهده شده بود. بنابراین نیاز به بررسی بیشتر در خصوص سبب‌شناسی این نوع از فلج مغزی در جامعه ما مطرح می‌گردد.
